# Qualitative analysis of Iranian sixth five-year economic, social, and cultural development plan from universal health coverage perspective

**DOI:** 10.1186/s12913-021-06985-1

**Published:** 2021-09-14

**Authors:** Mahdi Mahdavi, Haniye Sadat Sajadi

**Affiliations:** 1 The Bernard Lown Scholar in Cardiovascular Health, Harvard T.H. Chan School of Public Health, Boston, USA; 2 National Institute for Health Research, Tehran University of Medical Sciences, Tehran, Iran; 3 Knowledge Utilization Research Center, University Research and Development Center, Tehran University of Medical Sciences, Tehran, Iran

**Keywords:** Universal Health Coverage, Financial Protection, Equity, Effective Coverage, Health System Building Blocks, National plan, Iran

## Abstract

**Background:**

This research analyzed the Sixth Five-Year Economic, Social, and Cultural Development Plan of the Islamic Republic of Iran (6NPD) to shed light on how the plan addresses the Universal Health Coverage (UHC).

**Methods:**

This research was a qualitative study. We systematically analyzed ‘Secs. 14 -Health, Insurance, Health & Women, and Family’ in the 6NPD. Through a content analysis, we converted this section into meaning units and coded them. Coding was guided through the conceptual framework ‘Six Building Blocks of Health System’ and the key principles of UHC.

**Results:**

Six themes and twenty-one subthemes were identified. The subthemes of *financing* include a fair and secured process of resource pooling, payment methods, revenue generation for the health sector, and a definition of a basic benefits package. The subthemes of *governance and leadership* consist of social insurance policies’ integration, compliance of providers, a designation of the Ministry of Health and Medical Education (MoHME) as the regulator and the steward of health resources, a payer-provider split, and stakeholders’ participation. The subthemes *of health workforce* emphasizes balancing the quality and quantity of the health workforce with populations’ health needs and the health system’s requirements. The subthemes of *health information system*s consist of the electronic health records for Iranians, information systems for organization and delivery functions, and information systems for the financing function. The subthemes of the *organization and delivery* consider improving effectiveness and efficiency of healthcare delivery, strengthening the family physician program and referral system, and extending the pre-hospital emergency system. Lastly, *access to medicine* focuses on the design and implementation of an essential drug list and drug systems for approving the coverage and provision of generic medicine.

**Conclusions:**

The 6NPD introduced policies for strengthening the 6 building blocks of the health system. It introduced policies to improve financing particularly resource pooling and the sustainability of financial resources. As mandated by 6NPD, centering the health system’s governance/leadership in MoHME may exacerbate the existing conflict of interests and provoke various arguments, which impede the enforcement of rules and regulation. The 6NPD is a step forward in terms of improving financial protection, yet several other policies need to be made to adequately meet the requirement of UHC regarding equity and effective coverage.

## Background

Expanding Universal Health Coverage (UHC) is a paramount goal of health systems [1]. Simply prioritizing interventions for UHC, pouring money at the UHC programs, or providing all people with free coverage for an unlimited number of services will not succeed as no country has money and other resources to provide such services free of charge in a sustainable manner [2]. Therefore, nations need to efficiently employ strategies and governance arrangements to achieve UHC’s main goals [3]. Achieving UHC will require understanding what arrangements are envisaged for protecting the poor against the risk of impoverishments and catastrophic expenditures, thereby improving equity [1]. Countries need to know where they stand in their paths toward UHC, prioritize equitability, plan strategically, and spend realistically [3, 4].

The world health report 2000 defines UHC as the “ability to use health services that individuals need and that those services are of quality that improves health status” [5]. This definition addresses three key objectives of UHC: access to services when needed, quality of service, and financial protection against the risk of disease [4, 6]. Access to services refers to services that are already covered and extending coverage to other services. Quality refers to the effectiveness of services for improving health related to a need. Quality consists of three concepts; awareness of health status and need for health services, utilization as being enabled through financial means, and the effectiveness of services used to improve health status. In this regard, quality indicates if the health problem is “under control”. Financial protection is measured through the share of costs in the form of out-of-pocket expenditure or immediate cash payment. Equity can be linked to service utilization, effective coverage, and financial protection [7].

UHC provides a way to frame health system strengthening efforts for improving health system building blocks [4]. The six building blocks consist of (i) service delivery, (ii) health workforce, (iii) health information systems, (iv) access to essential medicines, (v) financing, and (vi) leadership/governance [8]. In terms of financing health services, UHC prescribes strategies to (1) cover entire populations rather than only extremely poor, (2) fund healthcare by a mandatory enrolment in basic insurance schemes, (3) provide all populations with a benefits package, (4) design the mechanisms of risk sharing, (5) and establish effective mechanisms of payment to providers [4, 6].

Given the importance of UHC, it should be addressed by the head of state plans and strategies. In the Islamic Republic (IR) of Iran, the Health Vision (2005) and the General Health Policies (2014) are two primary legal documents in which achieving UHC is highlighted [9]. To operationalize these macro-level policies, the Sixth Five-Year Economic, Social and Cultural Development Plan of the Islamic Republic of Iran (6NPD) was developed [10]. The 6NPD was enacted in 2016 as the country law that defines frameworks for planning governmental functions in various sectors. This law defined national policies and policy means to fulfill policies and define performance targets for the social, economic, and health development in the country. To the best of our knowledge, there is no research examining if and how health system building blocks are addressed by this law to contribute to the country’s progress towards UHC. This research examines how UHC as a key target is framing health system building blocks in Iran.

## Methods

This research was a qualitative content analysis study. It systematically analyzed Section 14 “Health, Insurance, Health & Women, and Family” of the 6NPD to elicit key policies related to UHC in Iran. It considered the official release of the 6NPD in July 2016 for [10]. The document consists of 20 sections and 124 articles.

The plan’s contents were analyzed through four steps: decontextualization, recontextualization, categorization, and compilation [11]. Through decontextualization, we got an overview of the plan and identified meaning units, i.e., codes. The coding process was guided through the six building blocks of health system [8]. For recontextualization, we went through the text to ensure that all the dimensions of the text have been converted into codes. Through categorization, we condensed the text and classified codes into subthemes. We subsequently classified subthemes to the building blocks as themes. The fourth step was the writing-up process [11].

We followed guidance on best practice qualitative research to design, analyze, and interpret data [12]. The study question is supported by the conceptual frameworks for UHC and health system building blocks. To minimize biases and increase trustworthiness, two researchers analyzed the 6NPD. Researchers discussed the agreement on disputed items. The co-authors critically reviewed the manuscript.

## Findings

Findings are presented for 6 themes. These themes consist of healthcare organization and delivery, health workforce, health information systems, access to essential medicines, financing, and governance and leadership (Table 1).

**Table 1:** Analysis of the Sixth Five-Year Economic, Social and Cultural Development Plan of the Islamic Republic of Iran for the Health sector: extracted codes, subthemes, and themes

Theme	Subthemes	Code
Organization and delivery	Improving effectiveness and efficiency of healthcare delivery	Outsourcing health services delivery to non-governmental sector.
		Rationing of health services delivery according to user needs and organized in three levels of health services delivery network
		Design and implementation of clinical guidelines for improving healthcare delivery.
	Family physician and referral system	Family physician program as the basis for referral system
		Gatekeeping and controlling patient free transition between the health system levels.
		Implementing universal and comprehensive healthcare system to get all Iranians under coverage of the referral system.
	Stakeholder participation in service delivery	Petroleum Industry Health Organization extends its network of healthcare providers in oil-rich areas and offshore to compensate for the adverse health impacts of harmful operations.
		Large companies in mine industry should equip and extend its network of healthcare providers in operational areas to compensate the adverse health impacts of harmful operations.
	Extending pre-hospital emergency system	Founding independent organization for pre-hospital emergency system.
Health workforce	Human resource development to meet health needs	Health needs as a basis for the capacity of health workforce educational institutions and quality and quantity of health workforces.
		Ministry of Health and Medical Education adjusts the capacity of educational institutions based on local health needs and gaps.
		Dual practice of specialist physicians and dentists are only allowed in deprived areas.
	Human resource development to support health system’s strategies and structures	Human resources for health are educated and trained according to needs of referral system and family physician program.
		Ministry of Health and Medical Education provides health/medical graduates for provider networks of Social Security Organization.
Information	Electronic health records for Iranian populations	Electronic health records for all Iranians
	Information systems to facilitate or substitute brick-and-mortar health service delivery	Electronic health service delivery
		Information system for health centers
	Information systems for health financing function	Information system for Health insurance services
		National data warehouse of health insurance enrollees
		Strategic purchasing supported by online infrastructures
Access to medicines	Design and implementation of a system for medicine	Design and implementation of generic medicine
		Develop a list of (essential) medicines
	Design and implementation of a system for medicine	Design and implementation of national drug system
	Provision of generic medicine	Provision of generic medicines
Financing	Effective payment methods	Drug reimbursement only based on the approved list of generic medicine.
		Strategic purchasing of health services
		Purchasing based on clinical guidelines
		Pay for performance for outsourced health services.
	MoHME to define benefit package	Define benefit package of health services
	Fair and secured process of pooling	Prepaid arrangements
		Mandatory enrolment in basic health insurance
		To determine contribution based on income level.
		Secured process for contribution collection.
		State subsidies to pay for the premium of extremely poor.
	Revenue generation	10 % increase in costs of divorce cases and allocation of collected money to reimburse costs of before-marriage genetic examinations.
		Revenue generation through taxing harmful products.
Governance and leadership	Compliance to fee schedules	Punishment for not adhering to tariff schedules.
	Compliance to prohibiting the dual practice of medical doctors, pharmacists, and dentists	Prohibiting the dual practice of medical doctors, pharmacists, and dentists in public and private sectors simultaneously.
	Compliance of all providers and insurers to policies made by MoHME	Mandating adherence of providers to decisions of MoHME.
		All providers and insurers follow MoHME policies.
	Health resource stewardship	Centralized management of financial resources
		MoHME as financial manager of health resources
		Acquisition of Iran Health Insurance Organization by MoHME
	Social insurance policy integration	Unified insurance policies and procedures
		Standardize all insurance policies and procedures
		Standardize health benefit package
		Standardize health insurance premium calculation method
	Payer-provider split	Strategic purchasing while reducing the ownerships of health organizations by MoHME.
		Strategic purchasing while reducing the provision of care by MoHME.
		Outsourcing health system functions that have no governance features
	Stakeholder participation, responsibility, and management	Making oil and mine industries responsible for their likely harmful operations on health
		Ministry of Industries, Mines, and Trade determines retail prices of tobacco products for tax purposes
		Ministry of Industries, Mines, and Trade provides access to information on the import and production of tobacco products for Iranian National Tax Administration
		Ministry of Agriculture provides Iranians with healthy foods
		Ministry of Health determines the capacity and number of medical/healthcare undergraduate and postgraduate students of governmental and nongovernment educational institutions.

### Organization and delivery

The subthemes of the organization and delivery included improving effectiveness and efficiency of healthcare delivery, family physician and referral system, stakeholder participation in service delivery, and extending pre-hospital emergency system. The 6NPD introduced policies for extending or fine-tuning existing structures and infrastructures of health services delivery. The 6NPD emphasized clinical practice guidelines, family physician program, referral systems/gatekeeping, and effective distribution of tasks between three health system levels. The development of clinical practice guidelines is mandated so that health services are provided based on the best available evidence. The law pointed out building a comprehensive health system with a clear role of family physicians in the referral system. Family physicians are expected to be the basis for the referral system and as gatekeepers to control unlimited access to specialized care in tertiary care. Particularly, a policy is introduced to limit the overutilization of tertiary care when primary care or secondary care can effectively meet the demands. The 6NPD also mandated the Petroleum Industry Health Organization and the Ministry of Industries, Mines, and Trade to compensate for the likely harmful impacts of petroleum and mining operations on the population’s health by taking a greater role in providing health services in remote areas and offshores.

### Health workforce

The 6NPD introduced policies to balance the quality and quantity of the health workforce with population health needs, health system’s requirements for effective functioning, family physician program’s strategies, and referral system functions. These policies are categorized into two subthemes: health workforce development to meet health needs and health workforce development to support the health system’s strategies and structures. The law mandated MoHME to adjust the capacity of all medical universities and higher educational institutions by the national and local health needs. The 6NPD even allows the dual practice of specialist physicians and dentists in deprived provinces and communities. MoHME also provides health/medical graduates for the healthcare system of the Iranian Social Security Organization.

### Health information system

The subthemes of the health information system consisted of electronic health records for all populations, information systems for organization and delivery function, and information systems for financing function. The 6NPD mandated establishing an electronic health record for all Iranians. It also required MoHME to facilitate health services delivery through web solutions or to substitute the traditional brick-and-mortar structure of health service delivery with health information technologies. On the one hand, preparation should be made to deliver health services through electronic systems, online platforms, and health portals. On the other, information systems should be provided for health centers to facilitate health service delivery. A national online data warehouse of health insurance enrollees will be constructed to strengthen the financing function. Under this function, the law required preparation to run an online system for strategic purchasing of health services. Furthermore, for revenue collection and transparency of tax on tobacco products, the National Tax Organization would get online access to data regarding the import and distribution of tobacco products.

### Access to essential medicines

Subthemes for access to essential medicine addressed the design and implementation of a system for approving and inclusion of medicine and the provision of generic medicine. Under this theme, the 6NPD mandated designing and implementing generic medicine, designing and implementing a national drug system, and developing a list of medicines for reimbursement purposes.

### Financing

Subthemes for financing included the fair and sustainable process of resource pooling, effective payment methods, revenue generation, and defining a benefits package. The 6NPD introduced various measures for the fair and sustainable process of pooling. These measures consist of prepaid arrangements for basic social insurance, relying on the mandatory enrolment in a basic health insurance scheme, determining individual contributions based on income level through a means test, using health information, i.e., online access to the national tax system to track revenues collectible from harmful products, and mandating the government to allocate subsidies to pay for the premium of extremely poor when the means test indicated the person is eligible for the government subsidy. Effective payment methods prescribed strategic purchasing of health services from non-governmental providers, purchasing health services based on clinical guidelines, pay for performance for outsourced health services, and reimbursement for only drugs included in an approved list of generic medicine. Revenue generation comprises the use of state subsidies to pay for the premium of the extremely poor, tax on the import and production of tobacco products, and allotting a 10 % increase in the fee for handling divorce cases through the judiciary system to reimburse costs of before-marriage genetic examinations.

### Governance and leadership

The subthemes under governance and leadership included mandates for social insurance policy integration, compliance to fee schedules, compliance of medical specialists to practice in the public sector only when employed by the public sector, designating MoHME as the regulator, central policymaking body, and steward of health resources, payer-provider split, and attracting stakeholders to participate and bear responsibility for health. The 6NPD prescribed the integration of social health insurance policies. It indicates that health insurance organizations keep functioning with current organizational structures, but they should follow a similar set of rules and regulations for financing arrangements. Based on this, insurance policies and procedures will be unified between insurers. Insurance policies and procedures, e.g., premium calculation method, will be standardized. The law emphasized the compliance of all providers to fee schedules and stressed punishing noncompliance. It also highlighted that all insurers should comply with policies that MoHME makes for health insurance schemes. Another key regulatory function of MoHME that is noted in the 6NPD addresses the financial management of health resources. The law stipulates the centralized management of financial resources and designates MoHME as the steward of that centralized resource management system.

The 6NPD introduced measures to outsource activities of MoHME that have non-governance features. It mandated MoHME to take a greater governance role and move away from functions that involve ownership and doing business with healthcare. With the current structure of the healthcare delivery system, which MoHME mostly owns, the law highlighted strategic purchasing as a mechanism to purchase services from the non-governmental sector; meanwhile, MoHME outsources healthcare provision. The 6NPD also emphasized reducing the ownership of health organizations by MoHME.

## Discussion

The 6NPD introduced several key health policies for the six building blocks of the health system. It introduced policies for a fair and sustainable process of resource pooling, effective payment methods, revenue generation, and cost containment methods for health system financing. Based on the 6NPD, all Iranians must be enrolled in a basic health insurance scheme to assure universal financial protection for the entire population, rather than only for the extremely poor. The mandatory enrollment in an insurance scheme and premium collection from individuals working for an informal sector or unregistered economy [13, 14] require extensive provisions. To this end, vast investment in infrastructure and information technologies are needed to collect data or update available data. There is no penalties for not being enrolled in the basic health insurance schemes [15]. Other insurance arrangements provide the same benefit as a mandatory enrollment policy. For instance, when a person has not purchased an insurance policy, in the event of a need for acute care, a bedside health insurance plan provides coverage for inpatient care. Thus, implementing mandatory enrollment by adequate means is essential to make sure that people do not opt out of a basic insurance scheme as they see another less expensive safety net when they are sick.

The 6NPD introduced a means test to determine contribution based on the individuals’ financial capacity and that enrollment is not hampered by inability to pay the premium. The premium for the poor would be paid by the government. The introduction of the means test is a step forward for an equitable and sustainable financing process. Yet, it remains to determine who is poor or eligible for governmental subside. Proxy measures such as income, age, employment, being under the umbrella of an employed person, or having consistent support from another person are used in other countries [16]. Among these measures, income as a proxy measure of the means test would be subject to heated debate as there is no agreement on a poverty threshold based on the income level in the country.

The plan introduced at least two sub-articles to control costs incurred by basic health insurance funds. For this purpose, the plan mandated defining a basic benefits package of health services. By this, policymakers wanted to only reimburse the costs of essential services rather than to generously reimburse the costs of a wide set of services. Before 6NPD passes into the law, there was a benefits package that was developed based on a little evidence base, untransparent criteria for inclusion of services, and a weak decision-making process. The revised benefits package should therefore transparently include cost-effective services while considering the conflict of interests [17]. Another cost control strategy was the operationalization of a referral system under the primary care program that is planned to be led by family physicians. This system is supposed to provide universal access to primary healthcare services with preferences given to preventive care and involving care at the community and household levels.

The governance section of the plan supports UHC by emphasizing centralized policymaking, strategic planning, evaluation, accreditation, and supervision of healthcare planning and finance in MoHME. It proposed centralizing the management of health resources in MoHME with emphasis on prepaid arrangements. It also mandated MoHME to design a basic benefits package and to unify insurance policies and procedures. Centering the governance of the health system in MoHME provokes various arguments. The most notable is that the MoHME is the main provider of health services and policies, for instance, strategic purchasing mechanisms that will be developed through the MoHME, in a first step, affect its affiliated hospitals. This is a great conflict of interest that impedes the effective enforcement of rules and regulations imposed by MoHME. One study showed that a conflict of interests is the main barrier to strategic purchasing [18]. Yet, the implementation of strategic purchasing is constrained by a lack of knowledge and experience in this subject area [19].

About strategic purchasing, there exist critical issues. A payer-provider split is a prerequisite for strategic purchasing, which is not the case in Iran [20]. Strikingly, with the integration of the Iran Health Insurance organization into the MoHME by 6NPD, the lack of payer-provider split becomes even more obvious. The accreditation of hospitals that determines the qualification of hospitals and in turn a rate at which hospital services would be reimbursed, is not conducted by an independent body [21]. It is officially located in MoHME. From the cost containment perspective, if done by an independent body, pay for performance, (e.g., payment based on accreditation results) would lead to an improved quality of care. The effective functioning of strategic purchasing and greater control power on the financing side of health services has always been preferred as a mechanism to control total healthcare expenditures [22].

Iran has taken steps to tackle the issues of UHC in the past two decades; most requirements set by the 6NPD for the governmental agencies while being aligned with UHC sound right for the current time [15]. However, accomplishing UHC targets requires investment not only in providing access to health services through improved healthcare financing but also investment in health system strengthening [23] for improving effective coverage and equity [5]. Though effective coverage is at the core of UHC, the law does not directly consider the inadequate level of this indicator in health priority areas such as cardiovascular diseases, diabetes, and mental health. For instance, of patients with treated hypertension; only 39 % are under control [24] and of patients with diabetes, only 13 % have controlled hyperglycemia [25]. Therefore, the 6NPD could have been more receptive to priority health areas, including chronic conditions, cardiovascular diseases, diabetes, mental health.

Equity seems to be pursued through prohibiting dual insurance coverage, standard premium calculation methods, unified insurance policies and procedure, and standard benefits package. Standard benefits package contributes to *equality* by entitling everyone to an equal set of benefits, though reducing inequity requires arrangements for additional protection for high costly diseases such as chronic diseases. For example, under a standard benefits package, patients with chronic diseases are entitled to a set of services similar to those with no chronic disease, despite the fact that, patients with chronic conditions face greater financial risk due to increased use of services and higher out-of-pocket expenditures that they incur [26]. In an analogy, these individuals need a benefits package on par with individuals with greater support from the Iranian health system, such as patients with rare diseases or cancers.

Based on the plan, MoHME also requires to equip and improve health services offshore and in oil-rich areas using the resources and capacity of the Petroleum Industry Health Organization and the Mine Industry Organization to make them socially responsible for the adverse impact of petroleum and mining operations on health. The 6NPD is establishing social responsibility for harmful operations of industries. Therefore, while the plan introduced articles for taxing the import and production of tobacco products, the law requires to extend taxing to other industries such as sugar-sweetened beverages. Taxing harmful products is at the beginning stage as yet several other harmful products are traded while taking little responsibility for likely harmful impacts on health.

The 6NPD also introduced measures to improve efficiency through need-based human resource development. As the health workforce educational system of the country is mainly funded by public money, the goal is to address health gaps and the health system needs by making an efficient use of the educational system capacity. Policies proposed by the 6NPD are expected to balance the demand and supply of the health workforce by adjusting the capacity human resource development system by the job market.

About transparency and accountability, a long path remains to go. Access to tobacco tax data is an example of increasing accountability and transparency for industries that do harm to health. The 6NPD could even make better use of transparency to leverage other building blocks, for example, in the governance area to reduce dual practice or to increase compliance to fee schedules.

The plan also mandated developing health records for all Iranian and supported progress towards e-health. Furthermore, to control the misuse of health services, the plan proposed developing a national online data warehouse of health insurance enrollees to contain dual coverage by multiple insurance funds. It will limit unequal access to services and increase risk-sharing. Though given the capacity of health information technology (HIT) in improving efficiency and patient experience in 21st century, it was expected from 6NPD to introduce more advancements using HIT.

Similar to the 6NPD, financing of health services is markedly addressed by the national health policies and health insurance arrangements in Iran, e.g., the Health Transformation Plan (HTP). Those policies aimed to reduce the proportions of healthcare expenditures [27] and were a societal response to ever-limited access to expensive health services and medicines [28]. In the same vein, the policies that are introduced by the 6NPD are born by the socio-economic contexts and demands for improved access to health services from both public and long-existing priorities. The plan is a step forward, yet several steps need to be taken to adequately meet the requirement of UHC. Alike other national plans for development, the 6NPD in the health sector should be seen as a document that addresses time-specific concerns. It does not include all means required to accomplish the introduced targets as those means might have been addressed by other head of state documents.

This research aimed to shed light on how the six building blocks of the Iranian health system are framed by UHC. Hence, further research is needed to display how the key objectives of UHC e.g. equity are directly or indirectly addressed, and the extent to which each UHC objective is realized. Future research may use quantitative data and research methods and longitudinal study designs to measure achievements of 6NPD in addressing health needs, effective coverage, and financial risk protection. Future studies of 6NPD may consider this evaluation using indicators that are already developed for the evaluation of 6NPD in the health sector [29]. It is worth noting that many rulings, including the permanent rulings of national plans for development and the rulings of 6NPD in the health sector that support the implementation of 6NPD in the health sector, were not analyzed in this research.

## Conclusions

This study assessed the 6NPD from the UHC perspective using the contents of the plan that was enacted to a law by the parliament. The 6NPD introduced policies that partially address the objectives of UHC in terms of increasing the utilization of health services in relation to health needs and enhancing universal financial protection. Effective coverage is an area that remains untouched. The 6NPH introduced policies that help health sector financing be more sustainable, primarily through a mandatory enrolment of entire populations into the social health insurance schemes. To encourage rational use of services, the MoHME as the governing body of the health system will design a basic health benefits package to be adhered to by all basic insurance schemes. From a payment perspective, the plan mandated using strategic purchasing to increase the efficient use of resources. Other policies included unifying health insurance policies across schemes, compulsory adherence by all healthcare providers to a formal fee schedule, and prohibiting contracts that reimburse surcharges of health services on top of the formal fee schedule. And to create a supportive environment for UHC, the governance of the health sector should becentered on the MoHME.

## Data Availability

Data, which underlie the analyses and findings reported in this research, mainly belong to the section of ‘Health and Family’ in the Sixth Five-Year Economic, Social, and Cultural Development Plan of Iran. The Iranian Consultative Assembly (the Parliament of Iran) officially released this plan (in Persian) on 10th of April 2017. The plan is available at various websites including: https://policy.asiapacificenergy.org/node/3671 http://rrk.ir/Laws/ShowLaw.aspx?Code=13138.

## Abbreviations

MOHME: Ministry of Health & Medical Education
6NPD: Sixth Five-Year Economic, Social and Cultural Development Plan of the Islamic Republic of Iran
UHC: Universal Health Coverage
HTP: Health Transformation Plan
HSD: Head-of-State Documents
